# Tubercular tubo-ovarian cystic mass mimicking acute appendicitis: a case report

**DOI:** 10.1186/1752-1947-5-363

**Published:** 2011-08-10

**Authors:** Sami Akbulut, Zulfu Arikanoglu, Murat Basbug

**Affiliations:** 1 Department of Surgery, Diyarbakir Education and Research Hospital, 21400, Diyarbakir, Turkey

## Abstract

**Introduction:**

Female genital tuberculosis is a rare form of extrapulmonary tuberculosis. It is an asymptomatic disease usually diagnosed during the search for causes of infertility. However, it can present with a number of abdominopelvic symptoms. Herein we report a case of tubo-ovarian tuberculosis mimicking acute appendicitis.

**Case presentation:**

A 17-year-old single Turkish woman presented to our hospital with complaints of right lower quadrant abdominal pain, nausea, and vomiting. Her physical examination findings, ultrasonogram, and leukocyte count were consistent with acute appendicitis. A cystic mass (15 cm × 6 cm) was detected on the right tubo-ovarian structure by laparotomy. The mass was excised while the tubo-ovarian structures were preserved and the need for an appendectomy was avoided. No microbiological evaluation was performed. The histopathological examination of the cystic mass revealed a granuloma with central caseating necrosis surrounded by epithelioid histiocytes. The patient was treated with anti-tuberculosis therapy for six months. No recurrence was observed during a 10-month follow-up period.

**Conclusion:**

Genital tuberculosis should be considered in the differential diagnosis of right lower quadrant pain in women who live in tuberculosis-endemic regions.

## Introduction

Appendicitis is the most common general surgical emergency in developed countries. Because this condition can imitate other benign causes of acute right lower abdominal pain and is frequently mimicked by other pathologies, it presents a major diagnostic challenge to the clinician [1,2].

The worldwide incidence of histopathologically normal appendices in patients who present with clinically suspected appendicitis ranges from 7.7% to 54% [3]. In the majority of female patients with negative appendectomy results, one or more gynecological pathologies are detected by histopathology or laparotomy. A variety of gynecological disorders can mimic acute appendicitis, including pelvic inflammatory disease (PID), ovarian torsion, tubal ectopic pregnancy, hemorrhagic cysts, recent ovulation, tubo-ovarian masses, or infected cysts, as well as granulomatous causes such as actinomycosis or tuberculosis (TB) [4].

TB remains an important public health problem worldwide [5,6]. The most common form is pulmonary TB, but the disease can affect almost any part of the body, including the lymph nodes, gastrointestinal tract, bone, retroperitoneal organs, vertebral structures, central nervous system, and genital tract [5,7]. TB is divided into two clinical forms: pulmonary TB (PTB) and extrapulmonary TB (EPTB). In African and South Asian countries with a high incidence of HIV infection, a significant increase in EPTB cases, particularly abdominopelvic TB, has been observed [8]. Genital TB (GTB) is a form of EPTB that occurs more frequently in women, in whom it classically presents in association with infertility, menstrual irregularity, or abdominopelvic pain involving the right lower quadrant [6,9]. In this case report, we present the case of a patient with genital TB that clinically mimicked acute appendicitis and caused the development of a tubo-ovarian cystic mass.

## Case presentation

A 17-year-old single Turkish woman presented to our emergency clinic with complaints of nausea, vomiting, and abdominal pain localized in the right lower quadrant. She stated that the localized pain had started three days prior to admission. The patient and her family had no history of TB infection or contact with patients with TB. Direct abdominal and chest radiography revealed no abnormality, and an abdominal ultrasound was also normal. Her pelvic examination, including pelvic ultrasonography, could not be performed because her bladder was empty. Her laboratory investigations produced the following results: blood urea nitrogen 35 mg/dL (5-23), creatinine 1.02 mg/dL (0,6-1,2), and C-reactive protein 12 mg/L(0-0,5). Her blood cell counts were leukocytes 12,200/μL (4-11000), hemoglobin 12.6 g/dL (12-16), and platelets 408,900/μL (150-450,000). Her other serum parameters were within normal limits. The results of an enzyme-linked immunoassay for HIV were negative. Urine analysis showed no pyuria or hematuria, and the results of a urine human chorionic gonadotropin assay were negative. The patient was placed under observation because her physical examination revealed no abnormality except right lower abdominal tenderness. A second abdominopelvic ultrasonogram was acquired during the observation period, and the results led to the suspicion of retrocecal appendicitis. Therefore, we performed surgery.

**Figure 1:**
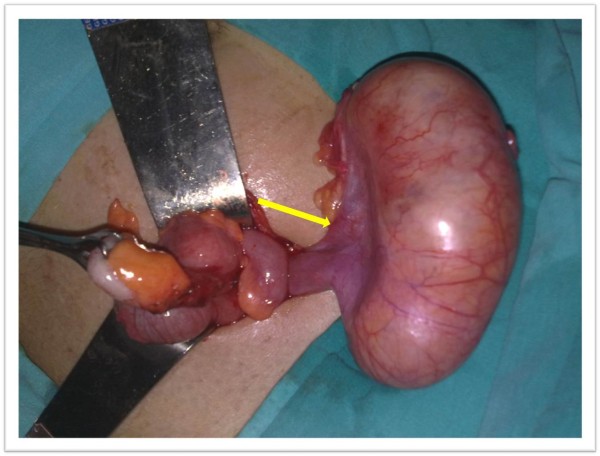
**A cystic mass, 15 cm × 6 cm in size, in an exophytic location and originating from the right tubo-ovarian complex (arrow)**.

**Figure 2:**
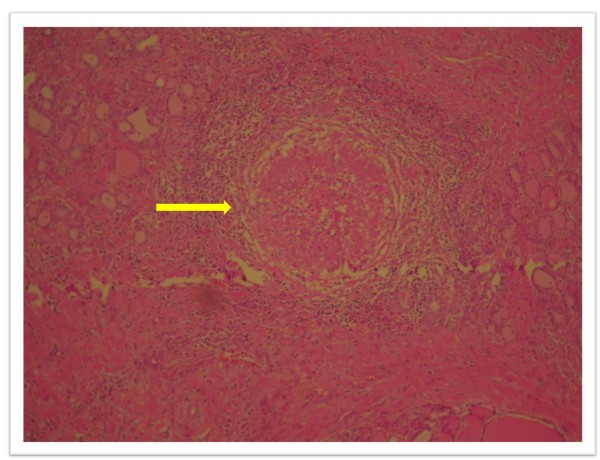
**Epithelioid granulomas (arrow) with central necrosis, surrounding epithelioid cells, some lymphocytes, and fibrosis at the periphery (original magnification, × 200)**.

**Figure 3:**
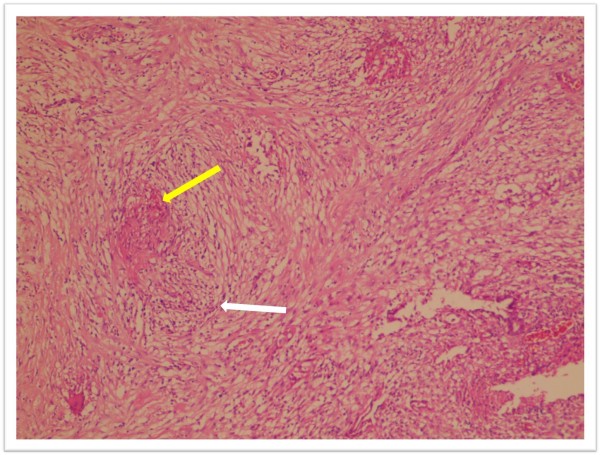
**Granulomatous inflammation process with central necrosis, epithelioid cells and lymphocyte halo at the periphery (white arrow)**. Scattered giant cells are present in the granuloma (yellow arrow) (original magnification, × 100).

A McBurney's incision revealed a cystic mass approximately 15 cm × 6 cm in size with regular margins. The mass originated from the right tubo-ovarian complex. The patient's appendix was clinically normal (Figure 1). The cystic mass was excised, and we preserved the tubo-ovarian complex and avoided the need for an appendectomy. When the cyst wall was cut, purulent liquid leaked through it. Microbiological evaluation of bacterial, fungal, and mycobacterial cultures and stains was not performed because the operation was performed over the weekend. The patient recovered fully and was discharged on the third post-operative day. Histopathological examination of the cystic mass revealed a granuloma with central caseating necrosis surrounded by epithelioid histiocytes (Figures 2 and 3). During the post-operative examination, we found a blood β-human chorionic gonadotropin level of 0.5 IU/mL, tuberculin skin test (the purified protein derivative test) 31 mm, and negative acid-fast bacillus (AFB) results of the urine and sputum tests. After the diagnosis was histopathologically confirmed, the patient was started on two months of anti-TB therapy consisting of daily doses of isoniazid (400 mg), rifampicin (600 mg), pyrazinamide (2 g), and ethambutol (1000 mg), followed by four months of isoniazid and rifampicin therapy. The total treatment duration was six months, and the patient recovered completely and without complications.

## Discussion

Acute appendicitis remains the most frequent cause of emergency gastrointestinal surgery. Its diagnosis is based on well-established clinical symptoms, basic radiological findings, and the surgeon's experience [1]. While most patients experience pain localized in the right lower quadrant, laparotomy reveals a histopathologically normal appendix in some cases. Hence, many medical and surgical factors other than appendicitis must be taken into consideration in the differential diagnosis of right lower quadrant pain. The differential diagnosis of appendicitis in women should include gynecological diseases such as cysts originating from the tubo-ovarian structures, abscesses, ruptured ectopic pregnancy, PID, and mittelschmerz, which is characterized by lower abdominal and pelvic pain that occurs roughly midway through a woman's menstrual cycle, particularly in women. Among these gynecological factors, PID and PID-related tubo-ovarian abscesses due to *Neisseria gonorrhea *and *Chlamydia trachomatis *bacteria are predominant. Chronic granulomatous diseases such as actinomycosis, fungal infection, and TB are rare and often have insidious causes. Actinomycosis usually develops in an ascendant manner as a result of long-term use of an intra-uterine device, whereas genital TB usually develops hematogenously and rarely by sexual contact [10-13].

According to the World Health Organization's 2009 report, one-third of the world's population is infected by TB bacilli and 9 million new cases occur each year [5,6]. EPTB is seen less often than PTB. EPTB may develop at an early stage of PTB or many years later. Lymphohematogenous dissemination from the pulmonary focus is most common, followed by intra-luminal and neighborhood spread. EPTB produces symptoms in the affected organ. Non-specific symptoms such as fever, night sweats, fatigue, and weight loss are more frequently observed in patients with PTB but also occur in EPTB cases. The absence of specific symptoms and conclusive signs during the physical examination may delay a proper diagnosis. Because of the differences in clinical symptoms, EPTB may be more difficult to diagnose than PTB. Thus, the disease should also be taken into account in patients with non-specific symptoms.

A form of EPTB, genital TB, affects about 12% of women with PTB and 15% to 20% of women with EPTB [6]. The actual incidence of genital TB among women are difficult to accurately determine because some women patients are asymptomatic and may remain undiagnosed. Primary GTB is an extremely rare genital tract infection that is almost always secondary to a focus elsewhere in the body [1,2]. The TB bacilli reach the genital tract by three principal routes. Hematogenous spread occurs in about 90% of patients, with the primary foci being the lungs, lymph nodes, and skeletal system [6]. Descending direct spread may also occur whereby the infection reaches the genital organs via the lymphatic system or directly from the gastrointestinal tract, mesenteric nodes, or peritoneum. TB bacilli are rarely transmitted by sexual intercourse. The fallopian tube constitutes the initial focus of genital TB in 95% to 100% of patients, followed by the uterus in 50% to 60% of patients and the ovaries in 20% to 30% of patients [6,12].

GTB has been described as a disease affecting women, with 80% to 90% of patients first diagnosed between the ages of 20 and 40 years [6,12]. The chief presenting complaints of women are infertility, vaginal bleeding, and chronic lower abdominal or pelvic pain [12].

Several risk factors exist for EPTB and TB of the female genital tract. Most are host factors causing impaired immunity, and increased exposure to the infection is also considered to be a risk factor. Diabetes mellitus, chronic renal failure, malignancy, HIV infection, long-term steroid use, alcoholism, and immunosuppressive therapy are also associated with an increased risk of EPTB [7,10,14].

The diagnosis of GTB is a clinical challenge and is rarely achieved by consideration of clinical symptoms alone, given their low specificity. A proper diagnosis usually requires additional data derived from abdominopelvic ultrasonography, chest radiography, PPD skin tests, AFB staining, polymerase chain reaction analysis and/or histopathological evaluation, and specific cultures from intra-operative specimens, including invasive surgical procedures such as diagnostic laparoscopy. However, TB infection cannot be ruled out by negative results of these tests. Up to 92% of chest radiographs, over 90% of Ehrlich-Ziehl-Neelsen stains and even pathological examinations can be negative in patients with extrapulmonary TB infection [10]. Although microbiologic culture is a gold standard for the definitive diagnosis of TB, a negative culture does not exclude the diagnosis of genital TB, as low microbiological burden, history of mycobacterial treatment or exposure to quinolone anti-microbial therapy, or inadequate culture preparation could cause a false-negative test [15].

Serum cancer antigen 125 (CA-125) levels and high-molecular-weight glycoproteins have been used to monitor the response of genital TB to anti-TB treatment [5,16]. In general, TB should be taken into account in the differential diagnosis of malignancy in patients with pelvic masses, ascites, and CA-125 levels < 500 IU/mL.

Anti-TB treatment should be added to the treatment strategy, and that treatment should be guided by susceptibility testing if cultures are available. The standard course of treatment for genital TB is isoniazid, rifampicin, pyrazinamide, and ethambutol for two months, followed by isoniazid and rifampicin alone for another four months [5,17].

According to our experience, in isolated cases wherein GTB that did not cause adhesion of surrounding tissues was detected, close follow-up without any anti-TB treatment has often been sufficient as long as the patient does not have a history that indicates susceptibility to TB, positive microbiological tests, or night sweats. However, in patients examined because of infertility who are diagnosed with GTB, anti-TB treatment may be given, regardless of the adhesion condition [6-10]. Although no complete consensus about this subject exists, studies are in progress. In the case of the patient we describe in this report, despite the absence of culture data, empiric TB treatment with four months of anti-mycobacterial therapy was initiated on the basis of the high endemicity of TB in the region, the presence of granulomatous histopathology, positive tuberculin skin test and risk for infertility if untreated TB.

In conclusion, GTB must be taken into account in the differential diagnosis of acute appendicitis, particularly in women of reproductive age who live in or are descendants of families from countries where TB is endemic and who have a history of chronic pelvic pain, menstrual abnormalities, and infertility.

## Abbreviations

EPTB: extrapulmonary tuberculosis; GTB: genital tuberculosis; PTB: pulmonary tuberculosis; TB: tuberculosis.

## Consent

Written informed consent was obtained from the patient's mother for publication of this case report and any accompanying images. A copy of the written consent is available for review by the Editor-in-Chief of this journal.

## Competing interests

The authors declare that they have no competing interests.

## Authors' contributions

SA, ZA, and MB analyzed and interpreted the patient data regarding the tubercular tubo-ovarian mass and the surgical findings. All authors read and approved the final manuscript.
